# Gene Cloning, Expression and Enzyme Activity of *Vitis vinifera* Vacuolar Processing Enzymes (*VvVPEs*)

**DOI:** 10.1371/journal.pone.0160945

**Published:** 2016-08-23

**Authors:** Yujin Tang, Ruipu Wang, Peijie Gong, Shuxiu Li, Yuejin Wang, Chaohong Zhang

**Affiliations:** 1 State Key Laboratory of Crop Stress Biology for Arid Areas, College of Horticulture, Northwest A&F University, Yangling, 712100, Shaanxi, China; 2 Key Laboratory of Biology and Genetic Improvement of Horticultural Crops (Northwest Region), Ministry of Agriculture, P.R. China, Yangling, 712100, Shaanxi, China; USDA-ARS, UNITED STATES

## Abstract

Vacuolar processing enzymes (VPEs) have received considerable attention due to their caspase-1-like activity and ability to regulate programmed cell death (PCD), which plays an essential role in the development of stenospermocarpic seedless grapes ovules. To characterize VPEs and the relationship between stenospermocarpic grapes and the *VPE* gene family, we identified 3 *Vitis vinifera VPE* genes (*VvβVPE*, *VvγVPE*, and *VvδVPE*) from the PN40024 grape genome and cloned the full-length complementary DNAs (cDNAs) from the ‘*Vitis vinifera* cv. Pinot Noir’ and ‘*Vitis vinifera* cv. Thompson Seedless’ varietals. Each of the VPEs contained a typical catalytic dyad [His (177), Cys (219)] and substrate binding pocket [Arg (112), Arg (389), Ser (395)], except that Ser (395) in the VvγVPE protein sequence was replaced with alanine. Phylogenetic analysis of 4 *Arabidopsis thaliana* and 6 *Vitis vinifera* VPEs revealed that the 10 *VPE*s form 3 major branches. Furthermore, the 6 grapevine *VPE*s share a similar gene structure, with 9 exons and 8 introns. The 6 grapevine *VPEs* are located on 3 different chromosomes. We also tested the enzymatic activity of recombinant VPEs expressed in the *Pichia Pastoris* expression system and found that the VvVPEs exhibit cysteine peptidase activity. Tissue-specific expression analysis showed that *VvδVPE* is only expressed in flowers, buds and ovules, that *VvγVPE* is expressed in various tissues, and that *VvβVPE* was expressed in roots, flowers, buds and ovules. The results of quantitative real-time PCR (qRT-PCR) suggested that *VvβVPE* in seeded grapes increased significantly at 30 days after full-bloom (DAF), close to the timing of endosperm abortion at 32 DAF. These results suggested that *VvβVPE* is related to ovule abortion in seedless grapes. Our experiments provide a new perspective for understanding the mechanism of stenospermocarpic seedlessness and represent a useful reference for the further study of VPEs.

## Introduction

Seedless grapes are classified into three types based on their pollination and fruit setting characteristics: spontaneous parthenocarpy, pseudoparthenocarpy and irritating parthenocarpy. At present, pseudoparthenocarpy seedless grape are the most commonly bred type because their seedlessness can be passed on to their progeny [1]. The flower structure of pseudoparthenocarpy grapes is normal, and their pollination and fertilization occur normally, but fertilized embryos undergo various degrees of ovule abortion, resulting in little to no perceptible trace of seed [2]. Furthermore, the ovule abortion trait of pseudoparthenocarpy grapes has value as a research tool because it is completely hereditary and is not influenced by various environmental factors [3].

Vacuolar processing enzymes (VPEs), which show caspase-1-like activity and regulate the programmed cell death (PCD), are cysteine peptidases in charge of the maturation of multifarious vacuolar proteins in higher plants. VPE was discovered in pumpkin seeds during their maturation [4]. Hara-Nishimura isolated and purified the enzyme from castor seeds and gave it the name VPE in 1991 [5]. VPE is synthesized from a larger pre-protein precursor (ppVPE) and then transformed into the mature form via autocatalysis. ppVPE contains a signal peptide (sp), an N-terminal propeptide (NTPP), a mature peptidase domain and a C-terminal propeptide (CTPP) [6]. The 55 kDa ppVPE is transformed into a 56 kDa precursor VPE (pVPE) in the endoplasmic reticulum through signal peptide removal and glycosylation, then transported into the dissolved vacuole, where it is transformed into a 43 kDa intermediate VPE (iVPE) via autocatalysis, and finally, into the 40 kDa mature VPE (mVPE) [6]. VPEs process proteins in the vacuole and show caspase-1-like activity to regulate PCD [7–8]. PCD pathways in animal are regulated by *caspases*. Once *caspases* are activated, they irreversibly trigger PCD [9]. *VPE*s are proved to be unique cysteine peptidases which pertain to a clade of the peptidase_C13 family by analyzing their molecular characterization [5]. Comparison of the amino acid sequences of human caspase-1, four *VPE* genes from tobacco, and *αVPE* and *γVPE* from *Arabidopsis thaliana* revealed that His and Cys in the catalytic dyad of VPEs are similar to two residues [His (237) and Cys (285)] in the catalytic dyad of human caspase-1. Furthermore, the important residues [Arg (179), Arg (341) and Ser (347)], which form substrate binding pocket of the human caspase-1, are conserved in VPEs [7]. There are four *VPEs*: *αVPE*, *βVPE*, *δVPE*, and *γVPE* in *Arabidopsis thaliana*. [10–11]. According to expression patterns and homology, they are classified into three types: vegetative type, seed type, and uncharacterized type. The distributions and functions of the three subfamilies vary. *αVPE* and *γVPE* are expressed in every tissue and belong to the vegetative type [12]. γVPE has been linked to plant senescence and stress physiology in apple, peanut, radish and *Arabidopsis thaliana* [13–20]. Many researchers found that *γVPE* deficiency enhanced the susceptibility of plants after pathogen invasion and demonstrated that γVPE shows caspase-like activity [21–23]. These results illustrated that γVPE participate in PCD regulation of plants. *βVPE* is expressed in seeds and take charge of the maturity of various proteins in seed storage vacuoles [24–26]. The expression of *δVPE* is highly specific and is observed only transiently during early development. It is considered an essential factor in the apoptosis of these two cell layers, which is significant during the forming process of seed coat [27].

VPEs are known to be involved in PCD [7–8], and embryo development in stenospermocarpic seedless grapes is dependent on PCD. These studies of *VPE* genes increase our understanding of the mechanism of seed abortion. In this study, we used *Arabidopsis thaliana VPE* sequences as queries to identify *VPE* genes in the grapevine genome and obtained 3 messenger RNA (mRNA) sequences. We then cloned 3 *VPE* full-length complementary DNAs (cDNAs) from both ‘*Vitis vinifera* cv. Thompson Seedless’ and ‘*Vitis vinifera* cv. Pinot Noir’, and the reverse transcription PCR (RT-PCR) products were sequenced and further analyzed using bioinformatics approaches. The expression profiles of *VvVPEs* were analyzed via quantitative real-time PCR (qRT-PCR) and semi-quantitative RT-PCR (sqRT-PCR) technologies. Finally, we tested the enzymatic activity of recombinant VPEs expressed in the *Pichia Pastoris* expression system. Our findings provide insight into the relationship between stenospermocarpic grapes and the *VPE* gene family and lay a foundation for further research of the agronomic traits of stenospermocarpic grapes.

## Materials and Methods

### Plant materials

‘*V*. *vinifera* cv. Thompson Seedless’, ‘*V*. *vinifera* cv. Youngle’, ‘*V*. *vinifera* cv. Pinot Noir’ and ‘*V*. *vinifera* cv. Flame Seedless’ grapevine plants were grown in the Grape Germplasm Repository of Northwest Agriculture and Forestry University Yangling, Shaanxi, China, under natural environmental conditions and standard management procedures. Embryos at 10, 15, 20, 25, 30, 35, 40, 45 days after full-bloom (DAF) of these four varieties were sampled and preserved using methods described previously [28]. Stem, root, leaf, tendril, alabastrum, flowers, pulp, and pericarp tissues were also sampled from ‘*V*. *vinifera* cv. Pinot Noir’ variety and treated as described above.

### Identification and isolation of grapevine *VPEs*

We utilized the *VPEs* available from the *Arabidopsis thaliana* Information Resource (http://www.arabidopsis.org/) as queries to search for grapevine *VPEs* in GenBank and subsequently employed the obtained sequences to scan the *Vitis* genome (http://www.genoscope.cns.fr/externe/GenomeBrowser/Vitis/) to confirm whether the sequences were redundant. Next, we queried the Pfam database (http://pfam.xfam.org/) to ensure that the sequences contained the Peptidase_C13 domain, the catalytic dyad (His and Cys), and the substrate pocket, which is composed of three crucial amino acids. The open reading frames (ORFs) of the *VPE* candidates were identified with the DNASTAR software. We designed primers for amplifying *VPE* genes by using the Primer premier 6.0 (S1 Table). The improved sodium dodecyl sulfate/phenol (SDS/phenol) method [29] was employed to isolate total RNA from ovules at each stage in four grapevine cultivars and from the stem, root, leaf, tendril, alabastrum, flowers, pulp, and pericarp tissues of ‘*V*. *vinifera* cv. Pinot Noir’. Synthesis of first-strand cDNAs and the cloning of *VPE*s’ full-length cDNAs were conducted with methods described previously [30]. We used ProtParam (http://web.expasy.org/protparam/) to calculate various parameters of the VPEs, including their molecular weight and isoelectric point (pI).

### Structures and positions of *VPEs* in grape chromosomes

The genomic DNA sequences and chromosomal locations of the *VPE*s were obtained through BLAST searches of each cloned sequences against the grape genome (http://www.Genoscope.cns.fr/externe/GenomeBrowser/Vitis/). The intron and exon organizations and predicted protein locations of the *VPEs* were analyzed using FGENESH-C (http://linux1.softberry.com/berryphtml?topic=fgenes_c&group=programs&subgroup=gfs).

### Phylogenetic analysis and multiple sequence alignments of grapevine VPEs

Multiple sequence alignment analysis of the putative amino acid sequences of 6 grapevine VPEs and 4 *Arabidopsis thaliana* VPEs was performed using ClustalX2 software. DNAMAN software was employed to analyze nucleotide sequence similarity of 6 *VPEs*. MEGA 6.0 software was used to build a phylogenetic tree of 6 grapevine VPEs and 4 *Arabidopsis thaliana* VPEs using minimum-evolution method with the WAG+G model, bootstrap method and 100 replications.

### Quantitative RT-PCR expression analysis of grapevine *VPEs*

Gene-specific primer pairs were designed for *VvVPEs* and each primer pair bound close to the 3’ untranslated regions (UTR) (S2 Table). The reactions were performed with the iQ5 real-time PCR machine (Bio-Rad, USA) and utilized SYBR Green I (TaKaRa Biotechnology, Cat.No.DRR041A). The reaction systems and conditions for qRT-PCR make reference to methods described previously [31]. iQ5 software was employed to analyze the relative expression levels of each *VPE*. Three technical replicates were performed to assess gene expression and *Actin* was used as an internal control gene.

### Semi-Quantitative Real-time RT-PCR expression analysis of grapevine *VPEs*

The cDNAs from stem, root, leaf, tendril, alabastrum, flowers, pulp, pericarp tissues and the mixed cDNAs of ovules at 8 developmental stages of ‘*V*. *vinifera* cv. Pinot Noir’ were used as templates. sqRT-PCR analyses were performed using a previously described method [30]. *Actin* was used as an internal control gene and the results were evaluated through agarose gel electrophoresis.

### Expression of recombinant VPE proteins in *Pichia pastoris*

*VvVPEs* were amplified with specific primer pairs (S3 Table). The *βVPE*, *γVPE*, *and δVPE* ORFs were inserted in the expression vector pPICZαA, and the recombinant plasmid were transformed into *Pichia pastoris* GS115 using lithium acetate/single-stranded carrier DNA/PEG (LiAc/SS-DNA/PEG) method. The positive colonies were identified and cultivated using methods described previously [32]. To induce expression, we added methanol every 24 h to maintain a final concentration of 0.75%. After 24 h, samples were collected every 12 h and the supernatant was collected as crude enzyme fluid after centrifugation at 3500 g for 5 min. Induction was stopped once the enzyme activity of the *VPE*s had been tested in the crude enzyme fluid (96 h). The supernatant was collected as crude enzyme fluid after centrifugation at 3500 g for 5 min. The crude enzyme fluid was freeze-dried into powder, and the powder was redissolved in double distilled water (ddH_2_O). The final protein concentration was quantified using Bradford method.

### Detection of VPE activity

The enzyme fluid was diluted to 0.1 mg/ml and then incubated with the same volume of reaction buffer [100 mM DL-Dithiothreitol (DTT) (Merck & Co Inc, Cat.No.233155), and 100 mM sodium acetate (NaAc) (Tianjin Damao Chemical Reagent Factory, Cas.No.127-09-3), pH5.5] for 8 h at 30°C. A 100 mM aliquot of the fluorescent *VPE*- specific substrate Ac-ESEN-MC (Acetyl-L-glutamyl-L-seryl-L-glutamyl -L-asparagine α-〈4-methylcoumaryl-7-amide〉) (Shanghai Botai Bio-Technique Co. Ltd., Cat.No.pep1604ESEN) was added to reaction buffer as described previously at a ratio of 1:40. The samples were wrapped in tinfoil and incubated for 5 h at 30°C. Fluorescence was measured using a previously described method [33].

## Results

### Identification, isolation, chromosomal locations and gene structure analysis of grapevine *VPEs*

**Fig 1:**
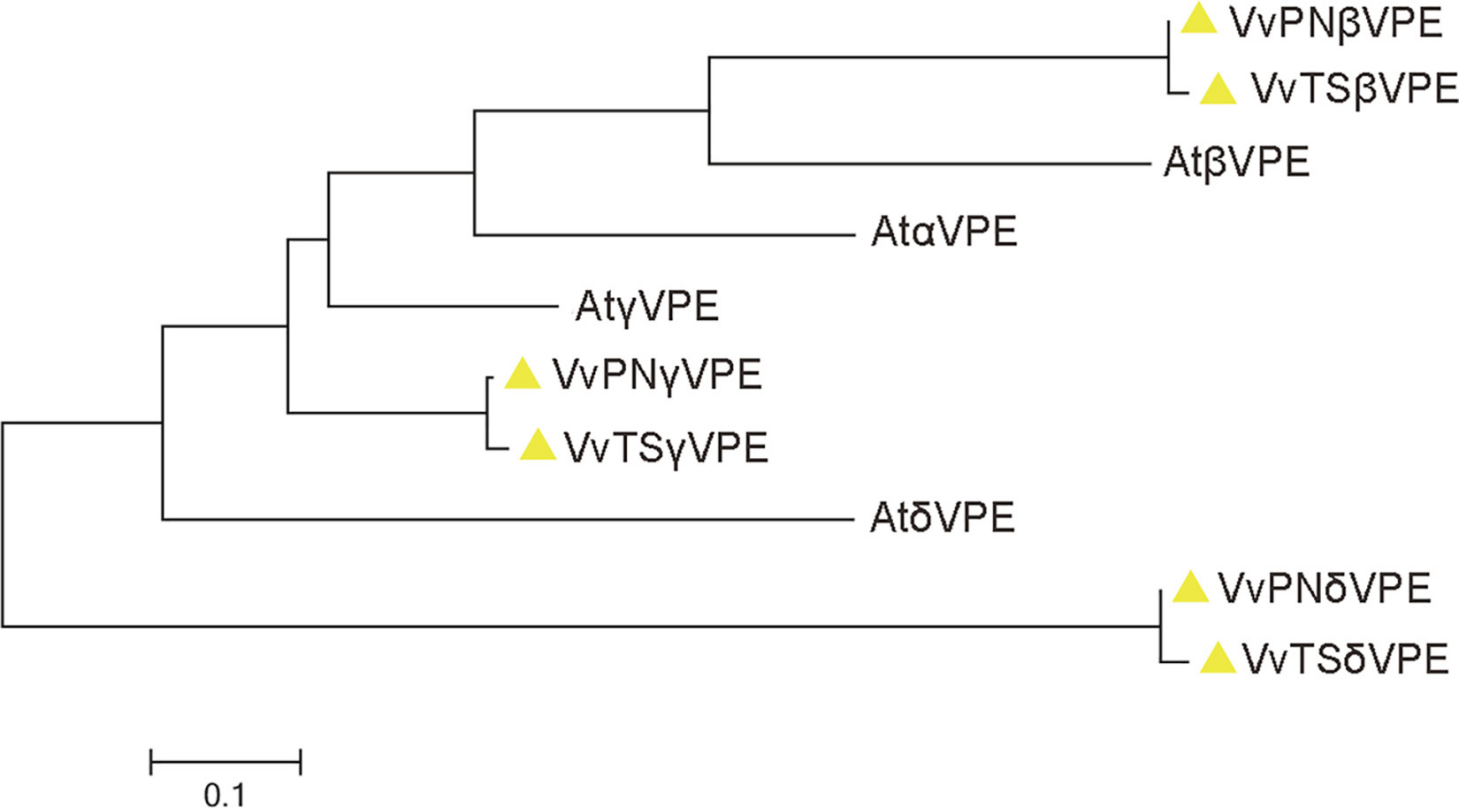
Phylogenetic analysis of grape and *Arabidopsis thaliana* VPE family proteins.

**Fig 2:**
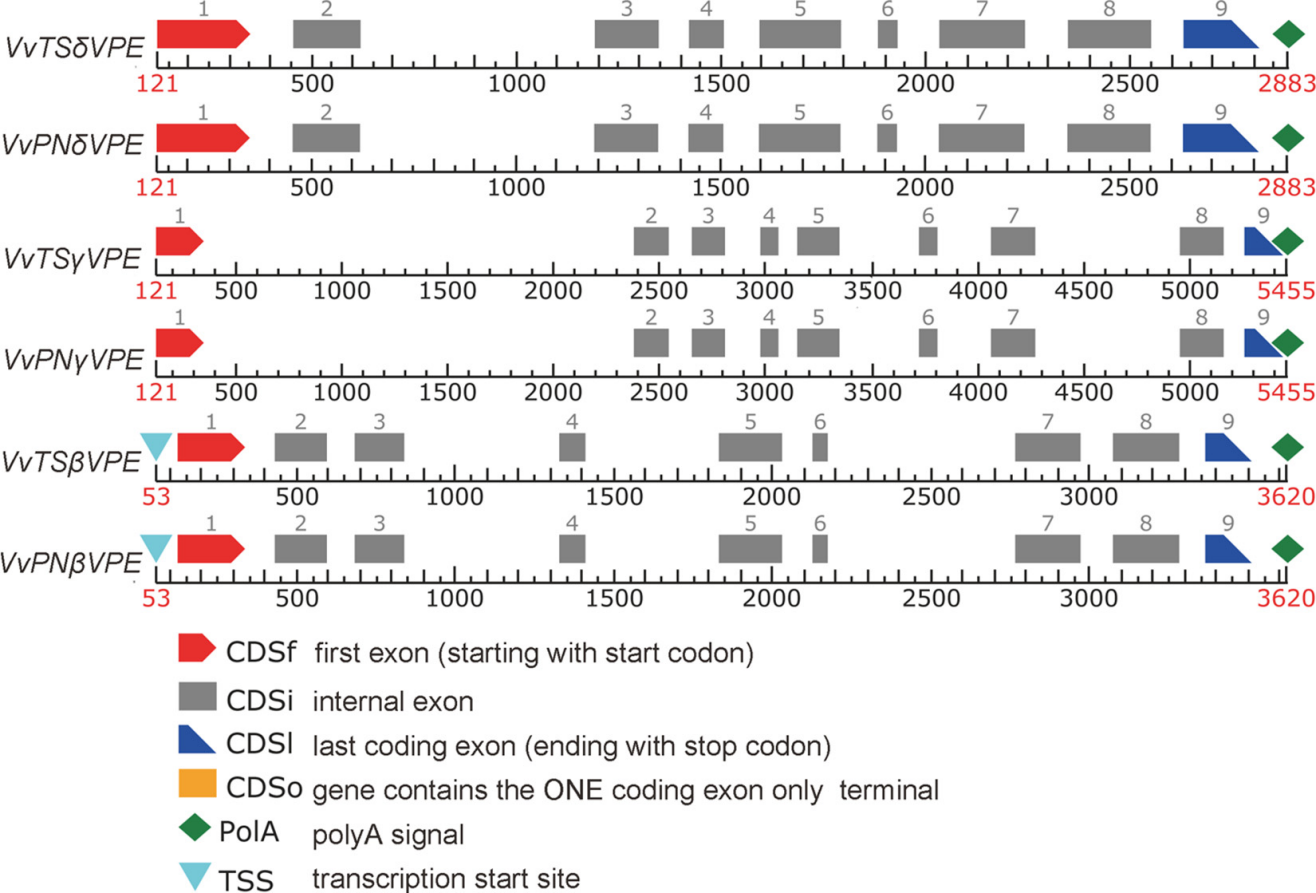
Intron and exon organization of *Vitis vinifera VPE* genes.

A total of 4 mRNA sequences were obtained. We found that XM_002265321.1 and XM_002263945.1 differed by 14 bases and 3 amino acids and that they were located at the same locus through a BLASTN search of the grape genome. Further examination of Expressed Sequence Tags (EST) and Transcriptome Shotgun Assembly (TSA) databases revealed that these two sequences were two predicted transcripts of a single gene. We queried the Pfam database with 3 putative VPEs and found that all of the sequences contain the Peptidase_C13 domain and belong to the *VPE* family. Thus, the grapevine genome contains 3 *VPE* family members, which we designated *VvβVPE*, *VvγVPE* and *VvδVPE* according to phylogenetic comparison with *Arabidopsis thaliana VPEs* (Fig 1) The full-length cDNAs of 6 identified *VPEs* were isolated from ‘*V*. *vinifera* cv. Thompson Seedless’ and ‘*V*. *vinifera* cv. Pinot Noir’ using RT-PCR approaches. The amino acid and ORF sequences of 6 VPEs have been submitted to GenBank: *VvPNβVPE* (KC136352.1), *VvPNγVPE* (KU240051), *VvPNδVPE* (KU240052), *VvTSβVPE* (KU240053), *VvTSγVPE* (KU240054), and *VvTSδVPE* (KU240055). Sequence alignment revealed that the similarity between these sequences and the predicted sequences was 99%. The length of the ORFs ranged from 1431 (*VvTSδVPE*) to 1485 bp (*VvTSβVPE*), and the length of the encoded polypeptides ranged from 477 to 495 aa, with predicted molecular masses ranging from 53.55 to 55.32 kDa. An analysis of the chromosomal loci of the 6 acquired sequences suggested that they were located on 3 different chromosomes (Table 1). The intron and exon organization of the grapevine *VPE*s was assessed, which revealed that all 6 *VPEs* exhibited a similar gene structure, consisting of 9 exons and 8 introns (Fig 2).

**Table 1:** Gene analysis of *VPEs* in *Vitis vinifera*.

Gene Name	Chromosomes	Position	ORF Length (bp) (aa)	Isoelectric Point (pI)	Molecular Weight (kDa)	GenBank accession number
*VvPNβVPE*	ChrUn	23374629–23377319	1485(495)	5.495	55.32	KC136352.1
*VvPNγVPE*	Chr4	641507–646819	1482(494)	5.290	53.89	KU240051
*VvPNδVPE*	Chr9	21507464–21510849	1431(477)	5.881	53.55	KU240052
*VvTSβVPE*	ChrUn	23374624–23377316	1485(495)	5.495	55.32	KU240053
*VvTSγVPE*	Chr4	641507–646819	1482(494)	5.217	54.04	KU240054
*VvTSδVPE*	Chr9	21507464–21510849	1431(477)	5.881	53.55	KU240055

### Phylogenetic analysis and multiple sequence alignments of grapevine VPEs

**Fig 3:**
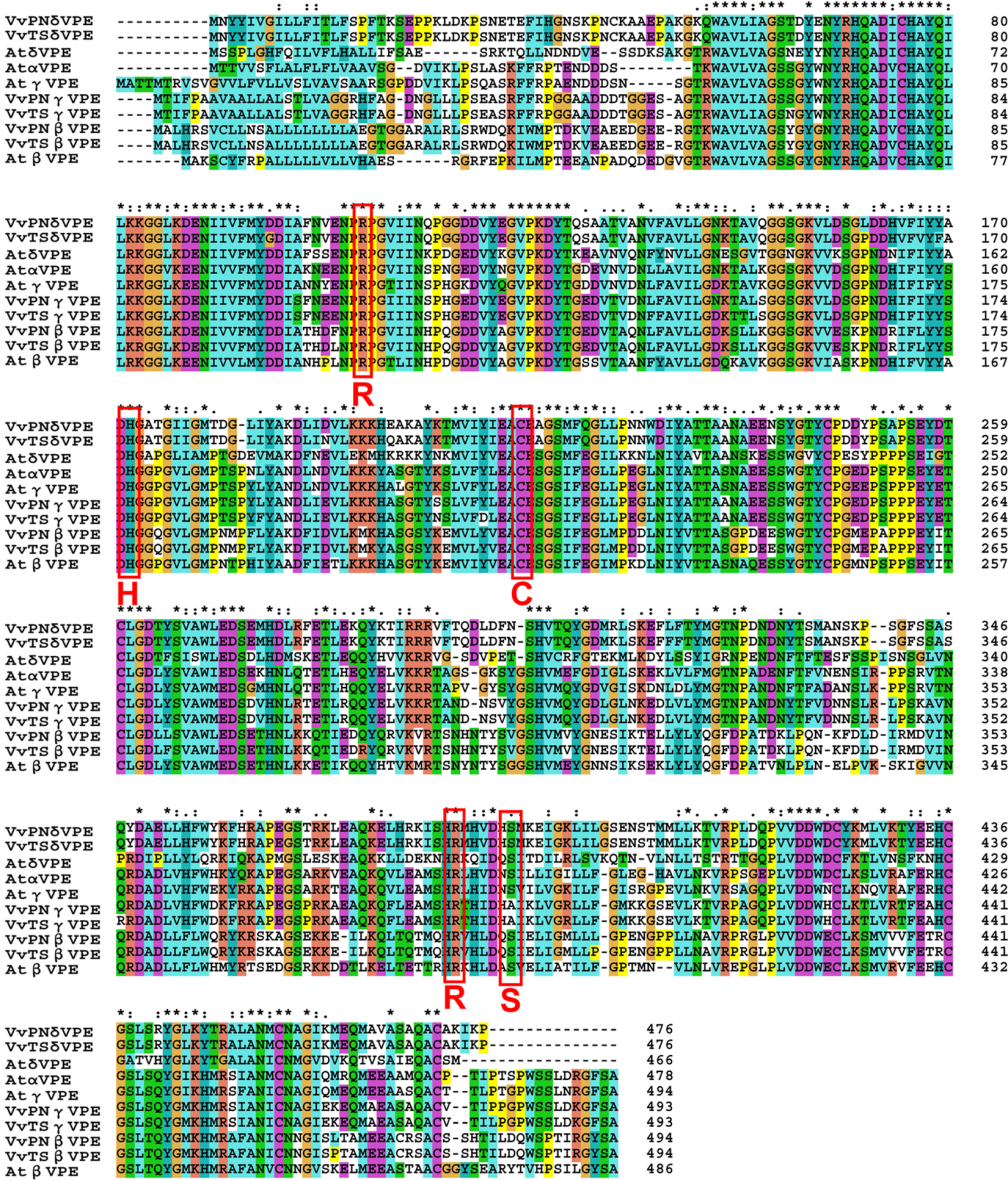
Multi-sequence alignment of *Vitis vinifera* and *Arabidopsis thaliana* VPE proteins. Amino acid sequences from 6 grapevine and 4 *Arabidopsis thaliana VPE* genes share similar catalytic dyad His (177), Cys (219) and the substrate binding pocket consisting of Arg (112), Arg (389), and Ser (395), with the exception of Ser (395) in VvγVPE, which was replaced with Ala (395).

The nucleotide sequence similarity matrix revealed that the similarity of the 6 *VvVPE* nucleotide sequences ranged from 46.5% to 58.3%. *VPE* genes from ‘*V*. *vinifera* cv. Thompson Seedless’ and ‘*V*. *vinifera* cv. Pinot Noir’ showed a relatively high similarity of up to 98% (S4 Table). In order to better understand the relationship between *VPE* gene family members, phylogenetic analysis and multiple sequence alignment were performed for the 6 grapevine protein sequences and the 4 known *Arabidopsis* VPE protein sequences (AtαVPE, AtβVPE, AtγVPE, AtδVPE). We found that each of the grapevine VPEs contained a stereotyped catalytic dyad of His (177) and Cys (219) as well as substrate binding pocket of Arg (112), Arg (389), and Ser (395), with the exception of Ser (395) in VvγVPE, which is replaced with alanine (Fig 3). The results of a phylogenetic analysis revealed that the 10 VPEs formed 3 large branches, and each branch contained VPEs from both grapevine and *Arabidopsis thaliana*. VvPNβVPE, VvTSβVPE, AtβVPE, and AtαVPE were classified as belonging to the first branch; VvPNγVPE, VvTSγVPE, and AtγVPE were grouped in the second branch; and VvPNδVPE, VvTSδVPE, and AtδVPE were grouped in the third branch (Fig 1).

### Expression analysis of grapevine *VPEs*

**Fig 4:**
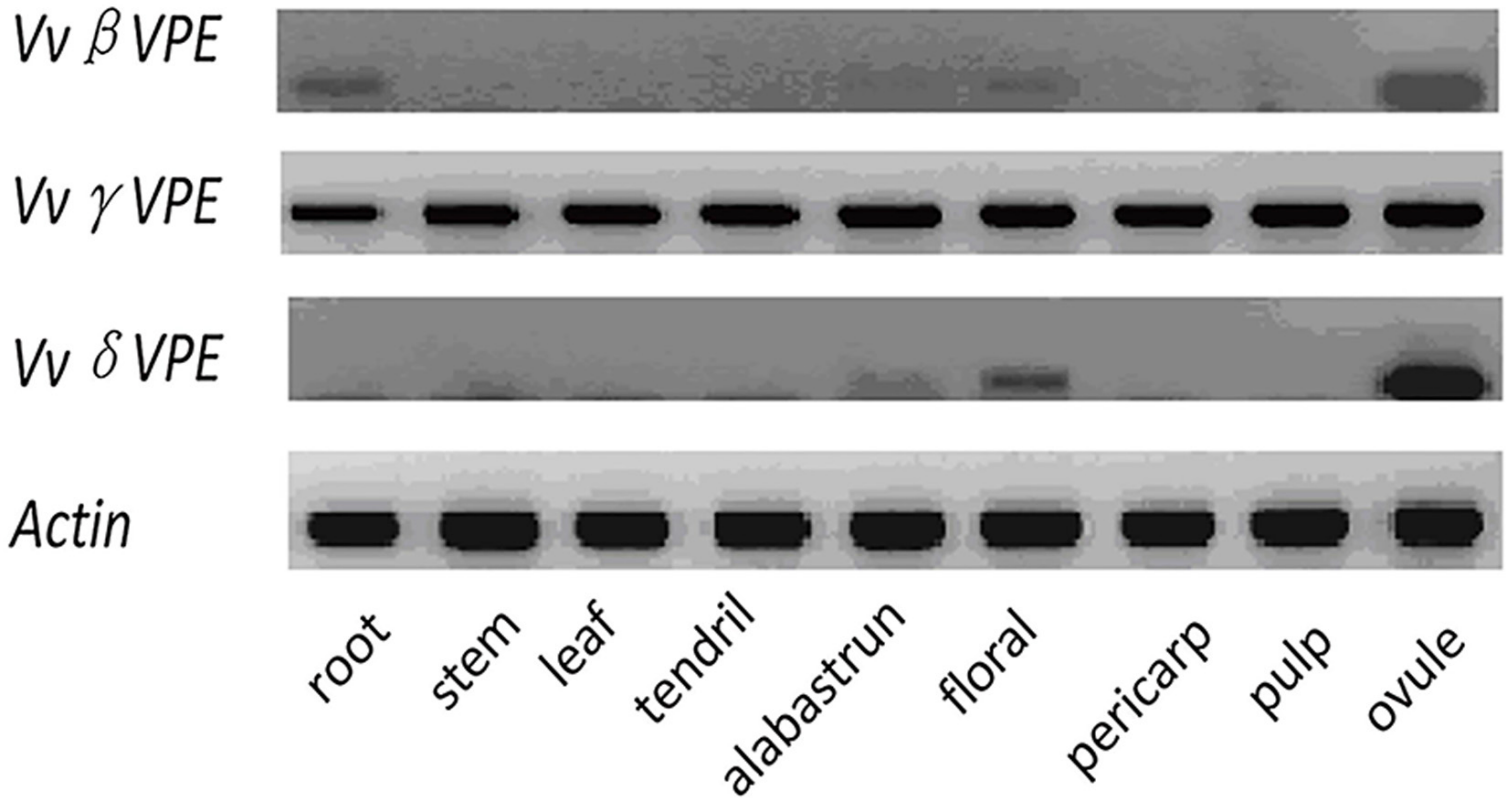
Expression of *VPE* genes in different grapevine tissues. Expression of *VPE* genes analyzed by sqRT-PCR in root, stem, leaf, tendril, alabastrum, flowers, pericarp, pulp, and ovule tissues of *Vitis vinifera* cv. Pinot Noir.

The expression of the grapevine *VPE*s in different tissues was assessed via semi-quantitive RT-PCR. We found that 3 grape *VPEs* exhibited distinct expression patterns in the examined tissues (Fig 4). *VvγVPE* was expressed in all tissues, whereas the expression of the other *VPEs* showed tissue and organ specificity to some extent. *VvδVPE* was expressed in the alabastrum, flora and ovules, and *VvβVPE* was specifically expressed in the root, alabastrum, flora and ovules.

**Fig 5:**
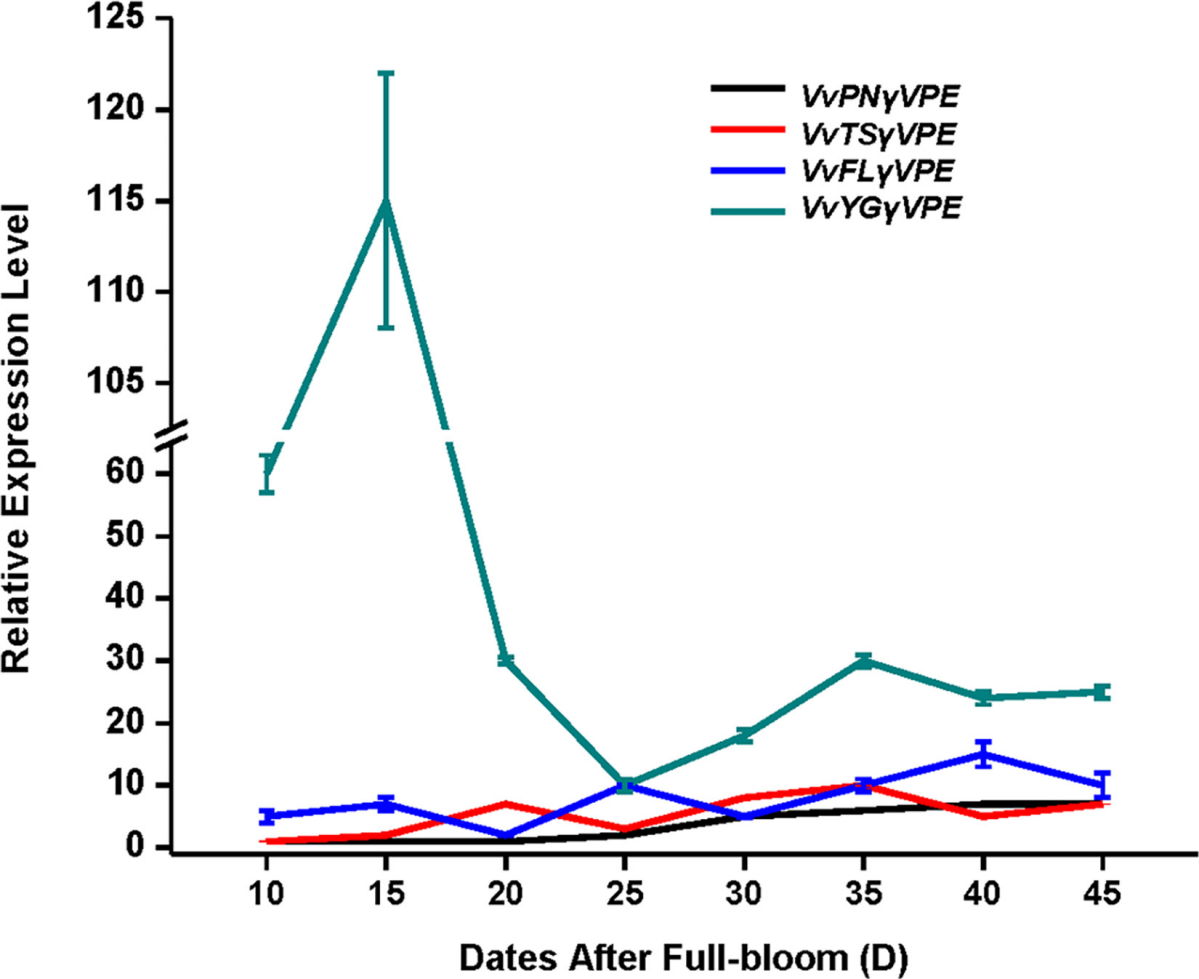
Expression of *VvγVPE* gene in different development stages of ovule in seed and seedless grapes. Relative expression level of *VvγVPE* gene in different development stages (15, 20, 25, 30, 35, 40, and 45 DAF) of ovule in ‘*V*. *vinifera* cv. Thompson Seedless ‘, ‘*V*. *vinifera* cv. Youngle’, ‘*V*. *vinifera* cv. Pinot Noir’ and ‘*V*. *vinifera* cv.Flame Seedless’. DAF (days after full-bloom) (error bars indicate ±SD).

**Fig 6:**
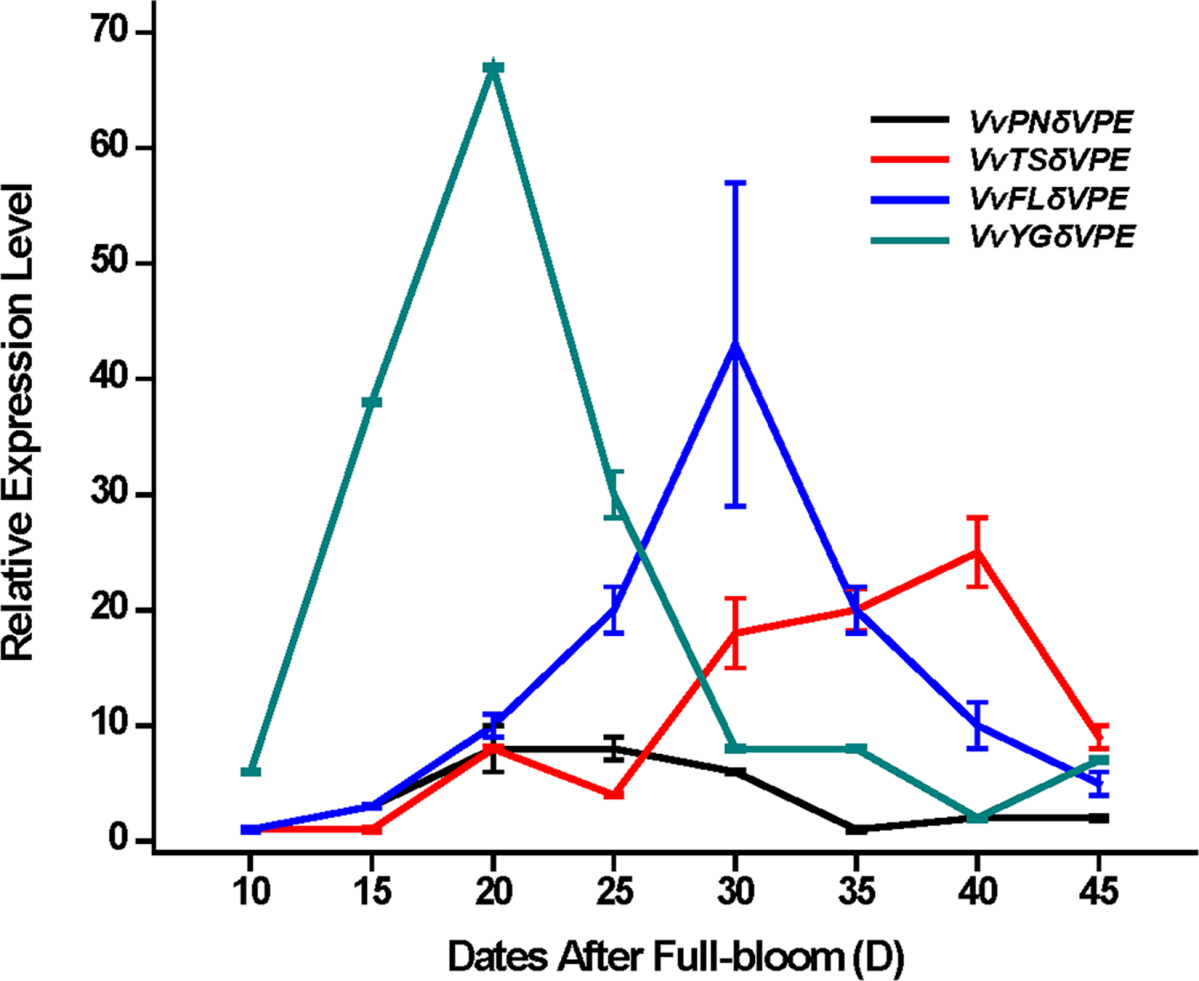
Expression of *VvδVPE* gene in different development stages of ovule in seed and seedless grapes. Relative expression level of *VvδVPE* gene in different development stages (15, 20, 25, 30, 35, 40, and 45 DAF) of ovule in ‘*V*. *vinifera* cv. Thompson Seedless ‘, ‘*V*. *vinifera* cv. Youngle’, ‘*V*. *vinifera* cv. Pinot Noir’ and ‘*V*. *vinifera* cv.Flame Seedless’. DAF (days after full-bloom) (error bars indicate ±SD).

**Fig 7:**
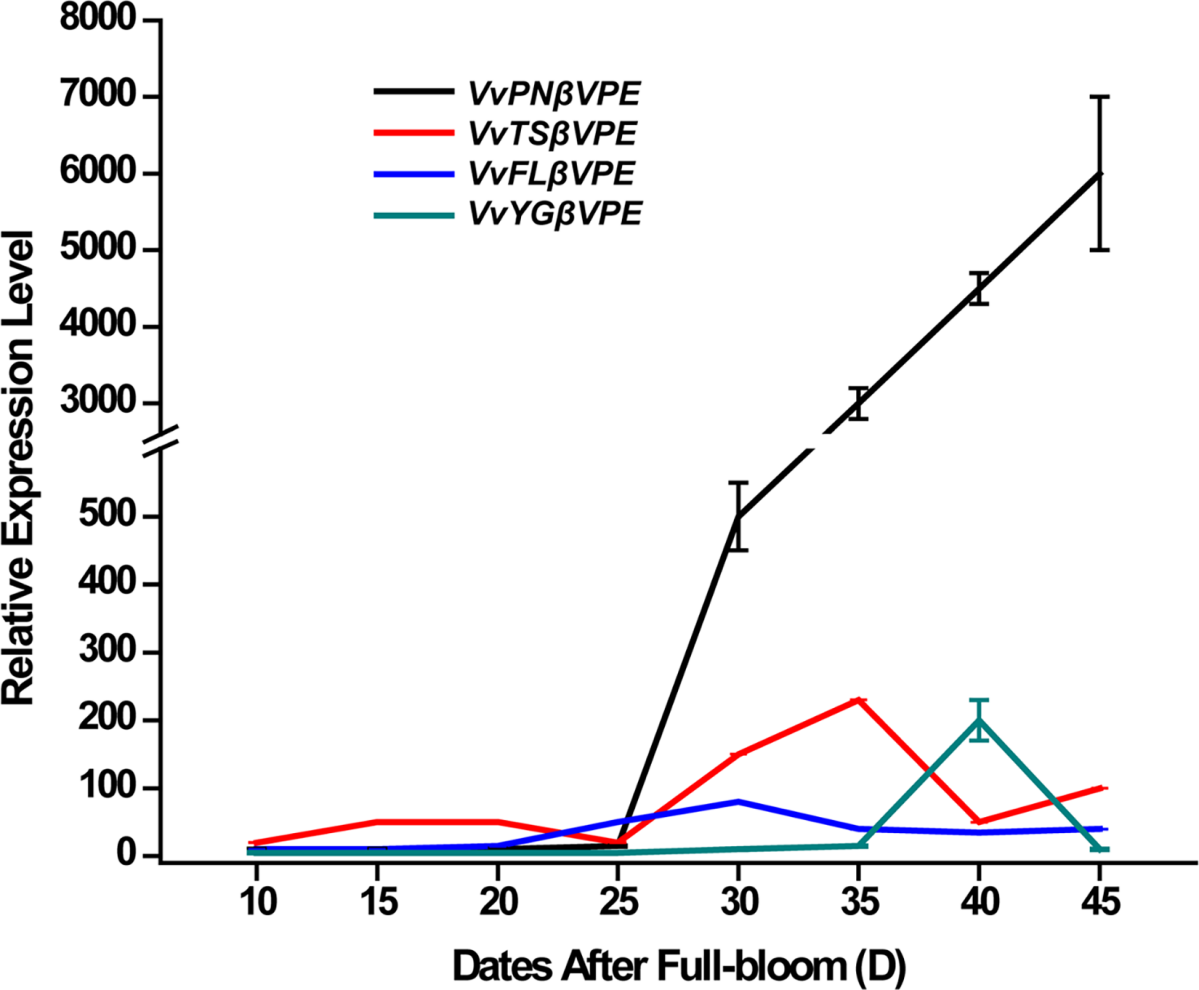
Expression of *VvβVPE* gene in different development stages of ovule in seed and seedless grapes. Relative expression level of *VvβVPE* gene in different development stages (15, 20, 25, 30, 35, 40, and 45 DAF) of ovule in ‘*V*. *vinifera* cv. Thompson Seedless ‘, ‘*V*. *vinifera* cv. Youngle’, ‘*V*. *vinifera* cv. Pinot Noir’ and ‘*V*. *vinifera* cv.Flame Seedless’. DAF (days after full-bloom) (error bars indicate ±SD).

qRT-PCR expression analysis of the grapevine *VPEs* revealed that *VvβVPE*, *VvγVPE* and *VvδVPE* have distinct expression profiles during the development of ovule between seeded (‘*V*. *vinifera* cv. Pinot Noir’) and seedless varieties (‘*V*. *vinifera* cv.Youngle’, ‘*V*. *vinifera* cv. Thompson Seedless’ and ‘*V*. *vinifera* cv. Flame Seedless’) (Figs 5–7). Furthermore, the expression of *VvβVPE* in these four grapevine varieties was relatively low during early development of ovule (approximately 10 to 25 DAF), and then the expression level in ‘*V*. *vinifera* cv. Pinot Noir’ increased sharply consistently until 45 DAF, whereas the expression levels in the ovules of ‘*V*. *vinifera* cv. Youngle’, ‘*V*. *vinifera* cv. Thompson Seedless’, and ‘*V*. *vinifera* cv. Flame Seedless’ were increased at approximately 40 DAF, 35 DAF, and 30 DAF respectively, and then drop back to the normal expression level. This finding revealed that *VvβVPE* gene exhibit different expression patterns between seeded and seedless grapevines. The result for *VvδVPE* showed that its expression in ‘*V*. *vinifera* cv. Youngle’, ‘*V*. *vinifera* cv. Thompson Seedless’, ‘*V*. *vinifera* cv. Pinot Noir’, and ‘*V*. *vinifera* cv. Flame Seedless’ shared a similar pattern, first being up-regulated and then recovered at approximately 20 DAF, 40 DAF, 20 DAF and 30 DAF respectively. The expression levels in seedless grapevines were higher than in seeded grapevine, but the difference was not significant. Thus, *VvδVPE* is not related to the stenospermocarpic seedless phenotype. The results for *VvγVPE* revealed that *VvγVPE* was also not related to the stenospermocarpic seedless phenotype. Expression in ‘*V*. *vinifera* cv. Pinot Noir’, ‘*V*. *vinifera* cv. Thompson Seedless’ and ‘*V*. *vinifera* cv. Flame Seedless’ was at a relatively low level. ‘*V*. *vinifera* cv. Youngle’ displayed significant up-regulation at approximately 15 DAF and then drop back to the same level as in the other three varieties.

### Detection of grapevine VPE enzymatic activity

**Fig 8:**
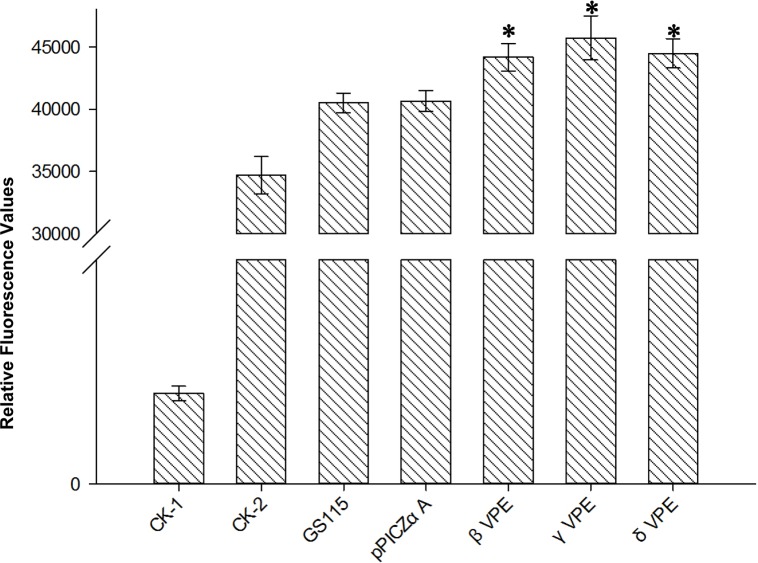
Enzymatic activity of grapevine VPEs. CK-1 (ddH_2_O); CK-2 (ddH_2_O and fluorescent VPE-specific substrate); GS115 (ddH_2_O, fluorescent VPE-specific substrate and GS115 thallus); pPICZαA (ddH_2_O, fluorescent VPE-specific substrate and GS115 thallus tansformed with pPICZαA plasmid); βVPE, γVPE and δVPE (ddH_2_O, fluorescent VPE-specific substrate and GS115 thallus transformed with recombinant plasmid). Enzymatic activity of recombinant grapevine βVPE, γVPE and δVPE were testified by using Ac-ESEN-MCA substrates at pH 5.5. Y-axes are scales of relative fluorescence values (error bars indicate ±SD).

To verify the enzymatic activity of the grapevine VPEs, we measured the enzymatic activity of crude enzyme fluid obtained from *Pichia pastoris* GS115 transformed with the *VPEs* of ‘*V*. *vinifera* cv. Pinot Noir’. The results (Fig 8) suggested that the relative fluorescence value of crude enzyme fluid obtained from *Pichia pastoris* GS115 transformed with the recombinant plasmids were approximately 4500, whereas the relative fluorescence value of the CK-1 (ddH_2_O) control group was extremely low. The value for CK-2 (ddH_2_O and fluorescent VPE-specific substrate) was approximately 3500. The relative fluorescence value of GS115 (ddH_2_O, fluorescent VPE-specific substrate and GS115 thallus) and pPICZαA (ddH_2_O, fluorescent VPE-specific substrate and GS115 thallus transformed with pPICZαA plasmid) were approximately 4000. These results showed that the relative fluorescence values of the crude enzyme fluid from *Pichia pastoris* GS115 transformed with recombinant plasmids were higher than those of the control group (P<0.05), indicating that *Pichia pastoris* GS115 transformed with grapevine VPEs secrete enzymes that could hydrolyze specific substrate; thus, the grapevine VPEs show cysteine peptidase activity.

## Discussion

### Characterization of *VPEs* in grapevine and the evolution of *VPEs* in plants

In an earlier study, members of *VPE* family were classified into vegetative-type *VPE*s and seed-type *VPE*s [34–35]. However, this classification does not apply to all *VPEs*. For example, Yamada found that *VPEs* from *Arabidopsis thaliana* could be divided into three subfamilies: vegetative-type *VPE*s, seed-type *VPE*s and new-type *VPE*s in 2005 [12]. Phylogenetic analysis was performed for 75 protein sequences from 22 genomes, including 3 VPEs from grapevine, which divided the genes into three subfamilies [36]. In this study, we identified three mRNA sequences, which we designated *VvβVPE*, *VvγVPE* and *VvδVPE*. The full-length cDNA sequences of 6 identified *VvVPEs* were isolated from the ‘*V*. *vinifera* cv. Thompson Seedless’ and ‘*V*. *vinifera* cv. Pinot Noir’ varieties using RT-PCR approaches. We found that 10 VPEs from *Arabidopsis thaliana* and grapevine formed 3 large branches, consistent with the classification scheme proposed by former researchers.

In parallel, the results of phylogenetic analysis and multiple sequence alignments revealed that the *VvVPE* genes from two grapevine cultivars were conserved and exhibited relatively high similarity. The existence of the *VPE* gene family is highly conserved among many different plants. For example, Yihua Wang and Kinoshita have found that *VPE* genes in both *Arabidopsis thaliana* and rice exhibit a 9 exon/8 intron structure [11, 37]. In the present study, we found that six acquired *VPE* sequences displayed this 9 exon/8 intron structure, providing additional support for the conservation of *VPE* among different plants.

A detailed structural analysis of 32 VPE proteins from rice, tobacco, and barley was carried out. Nishimura and Kinoshita found that all of these *VPE*s shared several structural properties with caspases, including the catalytic dyad of His (174) and Cys (285) as well as the substrate binding pocket comprised of Arg (179), Arg (341) and Ser (347) [8,12]. Multiple sequence alignment of the predicted protein sequences of 6 grapevine and 4 *Arabidopsis thaliana* VPEs showed that VvβVPE and VvδVPE contained the typical catalytic dyad of His (177), Cys (219) and the substrate binding pocket consisting of Arg (112), Arg (389), and Ser (395), implying that grapevine VvβVPE and VvδVPE are caspase-like peptidases. However, Ser (395) is replaced with alanine in the protein sequences of VvγVPE, and Ser (395) is one of the three important amino acids that form the caspase-1 substrate binding pocket. Nevertheless, our research demonstrated that VvγVPE shows cysteine peptidase activity. The results of phylogenetic analysis and multiple sequence alignments revealed that the organization of the *VPE*s in *Arabidopsis thaliana* and *Vitis vinifera* is very similar, indicating that all of the VPEs found in these groups might descended from a common ancestor.

### *VvβVPE* is linked to the seedless phenotype

In the pseudoparthenocarpy phenotype, the flower structure is normal, and pollination and fertilization occur normally. Thus, the seedless phenotype may due to the abortion of fertilized embryos during ovule development [2]. In higher plants, Proteins of seed are stored in protein storage vacuoles (PSVs) as a nutrients source that is necessary for seed germination, early growth, and development [38]. During the seed maturation process, storage proteins are synthesized in the rough endoplasmic reticulum as the form of precursors and then transported into PSVs via vesicles [39]. Subsequently, the storage proteins are hydrolyzed by peptidases at specific sites and transformed into their mature form prior to storage [25, 40–41]. Earlier research has demonstrated that βVPE plays a vital role in the processing of newly synthesized storage protein precursors. For example, in *Arabidopsis thaliana*, Shimada generated lots of mutants lacking different *VPE* isoforms. More than 90% of VPE activity disappeared in the *βVPE*-deficiency seeds, and a mass of storage protein precursors were found in the seeds. In contrast to *βVPE*-deficiency seeds, the other single and double mutants accumulated no protein precursors in their seeds. βVPE therefore plays a key role in processing of storage protein precursors [24]. In rice, a missense mutation in *OsVPE1* (a homolog of the *Arabidopsis thaliana βVPE* gene in *Oryza sativa*) that changes Cys (269) to Gly (269), leads to the accumulation of glutelin precursors, demonstrating that *OsVPE1* plays an essential role in the maturation of rice glutelins [37]. Toshihiro found that in the *OsVPE1* mutant, glutelin could not form the correct structure in protein storage vacuoles, which indicates that *OsVPE1* is important for the correct structure of storage proteins [42]. Phylogenetic analysis and multiple sequence alignment revealed that the acquired amino acid sequences of VvβVPE exhibit the typical catalytic active structure [His (177), Cys (219)] and a substrate binding pocket [Arg (112), Arg (389), and Ser (395)], which are similar to *Arabidopsis thaliana* VPE. Moreover, VvβVPE showed cysteine peptidase activity in our detection of enzymatic. Whether VvβVPE plays a crucial role in the processing of newly synthesized storage protein precursors just like AtβVPE and OsVPE1 must be further examined.

In this study, the grapevine *VvβVPE* gene was found to be specifically expressed in seeds and weakly expressed in roots. We found that *VvβVPE* was differentially expressed in later ovule developmental stages between seeded and seedless grapevines. In seedless grapevine, *VvβVPE* exhibited a relatively low expression level, whereas in seeded grapevine, *VvβVPE* increased significantly at 30 DAF, close to the timing of endosperm abortion at 32 DAF. VvβVPE showed cysteine peptidase activity, and the low expression level of *VvβVPE* in seedless grapevines could alter protein processing in vacuoles. All of these results suggested that VvβVPE is associated with ovule abortion in seedless grapes. Further studies are needed to reveal whether these expression changes hinder the development of grape ovules and affect normal ovules.

### Differential tissue-specific expression of *VPEs* among plants

In order to study the expression profiles of *VPE* genes from *Arabidopsis thaliana*, Kinoshita constructed three chimeric fusion genes composed of the coding region of the *β*-glucuronidase (gus) gene and the promoter region of each *VPE* gene [43]. This transgene was then introduced into *Arabidopsis thaliana* plants, resulting in the expression of *βVPE* in the seeds and the root tip [43]. However, sqRT-PCR analysis of *OsVPE1* revealed that *OsVPE1* transcripts were detected in all rice tissues, including the roots, seedlings, leaves, sheaths, shoots, flowers and endosperm [37]. In this study, the grapevine *βVPE* gene was observed to be specifically expressed in seeds and weakly expressed in roots. The expression pattern of grapevine *βVPE* is consistent with *Arabidopsis thaliana*.

Our sqRT-PCR analysis revealed that *VvγVPE* was expressed in all tissues and that the expression levels were equal. However, Kinoshita found that *γVPE* activity was not expressed in seed of transgenic *Arabidopsis thaliana* plants [43]. Exposure to pathogens, wounding and ethylene improved the expression of *γVPE*, which was involved in the suppression of stress [12, 43]. It is worth exploring whether *VvγVPE* shows the same reaction to wounding, ethylene and pathogens when it exhibits different tissue-specific expression patterns.

## Conclusion

In summary, we isolated 3 *VPEs*, designated *VvβVPE*, *VvγVPE* and *VvδVPE*, from the grapevine genome and cloned the full-length cDNAs from ‘*V*. *vinifera* cv. Thompson Seedless’ and ‘*V*. *vinifera* cv. Pinot Noir’. The finding that the 3 *VPEs* from grapevine formed 3 large branches was consistent with previous predictions from other researchers. In a eukaryotic expression system, it was found that *Pichia pastoris* transformed with the *VPEs* could secrete enzymes to hydrolyze specific substrates and that the VPEs from grapevines display cysteine peptidase activity. The expression profiles of *VvVPEs* over the course of ovule development between seedless and seeded grapevine and in different tissues are different. Notably, the grapevine *VvβVPE* gene is expressed specifically in seeds and shows a different expression pattern in the late development stage of the ovule between seeded and seedless grapevines. VvβVPE might be associated with the seedless grape ovule abortion process. Our experiment provides a new perspective for studying the mechanism underlying the stenospermocarpic seedless phenotype and represents a useful reference for the further study of VPEs. The main challenge in the future lies in understanding the role of VvβVPE during ovule abortion.

## Supporting Information

S1 Table Sequences of primers used to amplify *Vitis vinifera VPE* cDNAs. (DOCX)

S2 Table Sequences of primers used in *Vitis vinifera VPE* genes expression analysis. QACT-F and QACT-R are the primers of *Actin* as an internal control gene.
(DOCX)

S3 Table Sequences of primers used to amplify *Vitis vinifera VPE* cDNAs for cloning in pPICZαA vector. (DOCX)

S4 Table Sequence similarity matrix of 6 *Vitis vinifera VPE* nucleotide sequences. (DOCX)
