# Supplementary material for: Gene Cloning, Expression and Enzyme Activity of Vitis vinifera Vacuolar Processing Enzymes (VvVPEs)
Source: PLoS One. 2016 Aug 23;11(8):e0160945. doi: 10.1371/journal.pone.0160945 (PMC4994961; doi:10.1371/journal.pone.0160945)
Supplement: S1 Table — (DOCX) [file pone.0160945.s001.docx]

**S1 Table. Sequences of primers used to amplify *Vitis vinifera* *VPE* cDNAs**

| Primer | Primer sequence（5'→3'） | Primer | Primer sequence（5'→3'） |
| --- | --- | --- | --- |
| VvβVPE -F: | CTCTATGGCTCTGCATCGATCTGTT | VvβVPE -R: | TGAGGATCGGTCCAAATTGAGCT |
| VvγVPE -F: | ATGAACTACTACATTGTTGGCATAC | VvγVPE -R: | ATGGAAGATGATAATGGTAAG |
| VvδVPE -F: | CTCCTACCATTCCATTCGTCTC | VvδVPE -R: | TTGGGTATTGATTAGTCTCTGTG |
