# Supplementary material for: Gene Cloning, Expression and Enzyme Activity of Vitis vinifera Vacuolar Processing Enzymes (VvVPEs)
Source: PLoS One. 2016 Aug 23;11(8):e0160945. doi: 10.1371/journal.pone.0160945 (PMC4994961; doi:10.1371/journal.pone.0160945)
Supplement: S2 Table — QACT-F and QACT-R are the primers of Actin as an internal control gene. (DOCX) [file pone.0160945.s002.docx]

**S2 Table. Sequences of primers used in *Vitis vinifera* *VPE* genes expression analysis**

| Primer | Primer sequence（5'→3'） | Primer | Primer sequence（5'→3'） |
| --- | --- | --- | --- |
| *Q*VvβVPE-F: | GCAAACCCAACGACAGAATC | *Q*VvβVPE-R: | TAAATCATCAGGCATCAGACC |
| *Q*VvγVPE-F: | TTCTGAGGTTCTGAAGACTGTCCGGCCTGC | *Q*VvγVPE-R: | AGGCACTGAATCCCTTGTCAAGAGAGCT |
| *Q*VvδVPE-F: | AAGGGTATTTACTCAGGATCTGGATTTTAA | *Q*VvδVPE-R: | GCTGAAACCAGATGGCTTGGAGTTGG |
| Q*ACT*-F: | CTCTATATGCCAGTGGGCGTAC | Q*ACT*-R: | CTGAGGAGCTGCTCTTTGCAG |

QACT-F and QACT-R are the primers of Actin as an internal control gene
