# Supplementary material for: Gene Cloning, Expression and Enzyme Activity of Vitis vinifera Vacuolar Processing Enzymes (VvVPEs)
Source: PLoS One. 2016 Aug 23;11(8):e0160945. doi: 10.1371/journal.pone.0160945 (PMC4994961; doi:10.1371/journal.pone.0160945)
Supplement: S3 Table — (DOCX) [file pone.0160945.s003.docx]

**S3 Table. Sequences of primers used to amplify *Vitis vinifera* *VPE* cDNAs for cloning in pPICZαA vector**

| Primer | Primer sequence（5'→3'） | Primer | Primer sequence（5'→3'） |
| --- | --- | --- | --- |
| VvβVPE-EcoRI-F: | CGGAATTCTCTTCTCTATGGCTCTGCATCGA | VvβVPE-XbaI-R: | GCTCTAGAAGAGCACTATAACCTCTGATTGT |
| VvγVPE-EcoRI-F: | CGGAATTCCGATGACTATCTTTCCGGC | VvγVPE-XbaI-R: | GCTCTAGAAGGGCACTGAATCCCTTGTCA |
| VvδVPE-EcoRI-F: | CGGAATTCACATGAACTACTACATTGTTGGCAT | VvδVPE-KpnI-R: | GGGGTACCAGGGGTTTGATTTTGGCACA |
