# Supplementary material for: Gene Cloning, Expression and Enzyme Activity of Vitis vinifera Vacuolar Processing Enzymes (VvVPEs)
Source: PLoS One. 2016 Aug 23;11(8):e0160945. doi: 10.1371/journal.pone.0160945 (PMC4994961; doi:10.1371/journal.pone.0160945)
Supplement: S4 Table — (DOCX) [file pone.0160945.s004.docx]

**S4 Table. Sequence similarity matrix of 6 *Vitis vinifera* *VPE* nucleotide sequences**

| **Gene name** | ***VvPNγVPE*** | ***VvTSγVPE*** | ***VvPNβVPE*** | ***VvTSβVPE*** | ***VvPNδVPE*** | ***VvTSδVPE*** |
| --- | --- | --- | --- | --- | --- | --- |
| ***VvPNγVPE*** | 100% |  |  |  |  |  |
| ***VvTSγVPE*** | 98.2% | 100% |  |  |  |  |
| ***VvPNβVPE*** | 55.6% | 55.0% | 100% |  |  |  |
| ***VvTSβVPE*** | 55.2% | 54.6% | 98.8% | 100% |  |  |
| ***VvPNδVPE*** | 58.3% | 57.5% | 47.8% | 47.1% | 100% |  |
| ***VvTSδVPE*** | 57.7 % | 56.8% | 47.1% | 46.5% | 98.3% | 100% |
